# Impact of Food Environments on Obesity Rates: A State-Level Analysis

**DOI:** 10.1155/2023/5052613

**Published:** 2023-06-20

**Authors:** Elizabeth Cerceo, Elena Sharma, Anne Boguslavsky, Jean-Sebastien Rachoin

**Affiliations:** ^1^Cooper Medical School of Rowan University, Camden, New Jersey, USA; ^2^Division of Hospital Medicine, Cooper University Healthcare, Camden, New Jersey, USA; ^3^Department of Medicine, Providence Saint Peter Hospital, Olympia, Washington, USA; ^4^Division of Hospital Medicine, Lankenau Medical Center, Wynnewood, Pennsylvania, USA

## Abstract

**Introduction:**

Limited access to healthy food in areas that are predominantly food deserts or food swamps may be associated with obesity. Other unhealthy behaviors may also be associated with obesity and poor food environments.

**Methods:**

We calculated Modified Retail Food Environment Index (mRFEI) to assess food retailers. Using data collected from the Behavioral Risk Factor Surveillance System (BRFSS) survey, the NJ Department of Health (NJDOH), and the US Census Bureau, we conducted a cross-sectional analysis of the interaction of obesity with the food environment and assessed smoking, leisure-time physical activity (LPA), and poor sleep.

**Results:**

There were 17.9% food deserts and 9.3% food swamps in NJ. There was a statistically significant negative correlation between mRFEI and obesity rate (Pearson's *r* −0.13, *p* < 0.001), suggesting that lack of access to healthy food is associated with obesity. Regression analysis was significantly and independently associated with increased obesity prevalence (adjusted *R* square 0.74 and *p*=0.008). Obesity correlated positively with unhealthy behaviors. Each unhealthy behavior was negatively correlated with mRFEI. The mean prevalence for smoking, LPA, and sleep <7 hours was 15.4 (12.5–18.6), 26.5 (22.5–32.3), and 37.3 (34.9–40.4), respectively.

**Conclusion:**

Obesity tracks with food deserts and especially food swamps. It is also correlated with other unhealthy behaviors (smoking, LPA, and poor sleep).

## 1. Introduction

The rise in obesity rates over the past several decades has been abundantly studied. In general, the increase is attributed to consumption of calorie-dense foods and the widespread fall in physical activity across all ages. Low socioeconomic status neighborhoods have been disproportionately affected by changes in the food environment, with fast-food restaurants being overrepresented and health food stores being scarce [1]. The high levels of stress and food insecurity in low socioeconomic status (SES) communities are considered another contributing factor to the obesity epidemic [2]. On a national scale, the Center for Disease Control (CDC) reports the prevalence of obesity amongst US adults to be 42.4% in 2018; this number has been increasing since 1999 [3]. In New Jersey, specifically, the prevalence of adult obesity is reported to be 26%. New Jersey's overall rate of meeting adequate physical activity guidelines for adults is 49%, which is below the national average. Only 26% of adults get the recommended five servings of fruits and vegetables daily [3].

Previous studies have attempted to link the presence of food stores, including food deserts with body mass index ( BMI). However, the results have been inconsistent, indicating a more complex relationship between dietary habits, food accessability, environmental factors, and obesity [4]. For example, some studies suggest that lower price is significantly associated with obesity but not distance to food stores [5, 6]. Food displays and marketing have also been linked to consumption patterns. Food deserts have little in the way of healthy food options (supermarkets and health food stores) while food swamps flood the market with high-calorie food from convenience stores and fast-food restaurants [6, 7]. There is some evidence that residing in a food swamp has a greater impact on BMI compared to a food desert [8, 9]. Thus, both food deserts and food swamps may lead to more frequent purchasing of unhealthy food and higher BMI [10, 11]. Other studies show that while convenience stores are often maligned as selling calorie-dense snack foods that encourage unhealthy eating habits, interventions targeting these stores such as adapting social cognitive theory, social ecology, and systems theory to implement environmental changes which would prioritize placement of healthy food could increase healthy food stocking [12]. Multiple studies investigating specific regional trends in food availability and obesity have led to often discrepant findings [9, 13–19]. This heterogeneity of results may imply impacts of other local and regional factors on weight and food retail environments.

Along with unhealthy eating habits, there are other behaviors not conducive to health that may affect one another or signal the coexistence of other unhealthy behaviors. Lack of physical activity may be a contributing factor to obesity but is associated with poorer outcomes even in the absence of obesity [20–22]. Further clarifying factors associated with obesity can also identify when unhealthful behaviors track together and, while they do not imply causation, may suggest areas of future research as well as topics that should be addressed in clinical care of patients. The relationship of this and other health-related behaviors may impact obesity more profoundly than the local food environment.

Because of inconsistencies in the existing evidence based on the relationship between obesity and food environment, we aimed to analyze the relationship between food environments and public health focusing on New Jersey, a densely populated and demographically diverse state with an array of food environments. We additionally hypothesized that obesity rates were inversely proportional to the quality of food environment and proportional to negative health-related behavior.

## 2. Methods

### 2.1. Design

Using data collected from the Behavioral Risk Factor Surveillance System (BRFSS) survey, the NJ Department of Health (NJDOH), and the US Census Bureau, we conducted a cross-sectional analysis of the interaction of obesity with the food environment.

### 2.2. Definitions

#### 2.2.1. Census Tracts

Census tracts are contiguous subdivisions of counties with populations ranging from 1,200 to 8,000 [23].

#### 2.2.2. Modified Retail Food Environment Index

The CDC food environment rating system is based on the ratio of healthy food retailers compared to total food retailers in a geographic region. To quantify the food environment, we calculated Modified Retail Food Environment Index (mRFEI) by dividing the total number of healthy grocery stores in a geographical region by the sum of both healthy and unhealthy food retailers found in the same region [24].

(1) $$\begin{array}{c} mRFEI=100\;x\;\frac{\#healthy\;food\;retailers}{\#healthy\;food\;retailers+\#less\;healthy\;food\;retailers}. \end{array}$$

Supermarkets, larger grocery stores, supercenters, and produce stores are considered healthy food retailers as categorized by North American Industry Classification Codes (NAICS). Less healthy food retailers include fast-food restaurants, small grocery stores, and convenience stores.

The mRFEI represents the percentage that is healthy [25]. Population data used in this study were obtained primarily from the annual CDC's BRFSS survey [26]. We then calculated the mRFEI for each census tract by including retailers who are within a half-mile of the tract boundary. Each tract has a score ranging from 0 to 100, where a higher value corresponds to a higher quality food environment.

#### 2.2.3. Food Deserts and Food Swamps

A food desert is a census tract with mRFEI scores of zero (no healthy food retailers), as defined by the United States Department of Agriculture [27]. An mRFEI score of 1–4 is considered a food swamp.

#### 2.2.4. Obesity, Smoking, Sleeping, and Leisure-Time Physical Activity (LPA)

Obesity is a BMI equal to or greater than 30.0, as defined by the World Health Organization (WHO) [28]. Smoking is the crude prevalence of current smoking among adults aged ≥18 years, 2018. Sleeping is the crude prevalence of sleeping less than 7 hours among adults aged ≥18 years, 2018. LPA is the crude prevalence of no leisure-time physical activity among adults aged ≥18 years in 2018.

### 2.3. Data Acquisition and Matching

We used data from BRFSS 2018 survey, US Census Bureau 2010 population estimates, and American Community Survey (ACS) 2014–2018 or 2013–2017 estimates. We matched the census tract level, with a corresponding geolocation to the values from the mRFEI yielding 1,695 matched tracts within the state of New Jersey.

### 2.4. Statistical Analysis

We present categorical data with percentages and continuous data with median (Interquartile Range). We calculated Pearson correlations to relate responses of the BRFSS survey (behaviors such as smoking, sleeping, and exercise) to obesity prevalence and the mRFEI. The correlation coefficients are tested for statistical significance based on the *p*=0.05 (two-tailed). We additionally performed univariate and multivariate regression analyses. We analyzed univariate differences with the Mann–Whitney *U* test and Kruskal–Wallis tests. We then performed a linear logistic regression to assess the independence of the association between the combined factor “Food Swamp and Deserts” and the outcome of obesity prevalence. All factors were entered in one-step and kept if the *p* value was <0.1. All analyses were done in SPSS 28.0 (IBM, Chicago, IL).

## 3. Results

### 3.1. mRFEI, Food Deserts, and Food Swamps

Out of 2,010 census tracts in New Jersey, mRFEI data are available for 1,945 tracts. Of those, there is corresponding geolocation with BRFSS data for 1,695 tracts that were included in the study. Scores for mRFEI range between 0 and 100 for each track. The median mFREI was 9 [4–15]. We found that there were 17.9% food deserts and 9.3% food swamps in the state overall (total of deserts plus swamps, 27.2%). We present in Table 1 the results at the county level. The scores varied greatly from one county to another with the highest being in Mercer county (40.6% of tracts were food deserts and swamps) and the lowest in Cumberland county (11.1%).

#### 3.1.1. Obesity, Smoking, Sleeping, and Leisure and Pleasure Activity (LPA)

Obesity prevalence in the state was 27.9% (24.9–32.7). There were 15.4% smoking on average. The mean prevalence for smoking, LPA, and sleep <7 hours was 15.4 (12.5–18.6), 26.5 (22.5–32.3), and 37.3 (34.9–40.4), respectively.

### 3.2. Correlations Analysis

We found a statistically significant negative correlation between mRFEI and obesity rate with Pearson's *r* value of −0.13 (*p* < 0.001), suggesting that lack of access to healthy food is associated with obesity. Additionally, obesity correlates positively with the following unhealthy behaviors: smoking (0.839, *p* < 0.001), LPA (0.819, *p* < 0.001), and sleep (0.767, *p* < 0.001). Each of the unhealthy behaviors listed (smoking −0.186, LPA −0.223, sleep −0.229, all *p* < 0.001) was negatively correlated with mRFEI.

### 3.3. Food Deserts, Swamps, and Obesity

**Figure 1:**
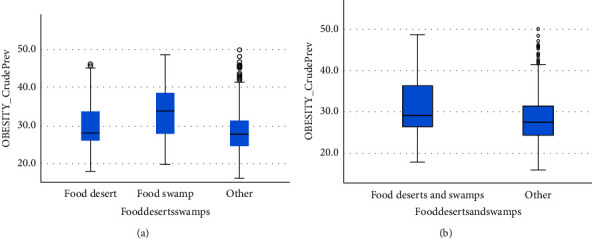
Obesity prevalence by census tract.

We found a significant difference between the prevalence of obesity in food deserts 28 (26–33.7), food swamps 33.8 (27.8–38.6), and other regions 27.6 (24.4–31.3) (Kruskal–Wallis, *p* < 0.001). There was a significant difference between the combined food deserts and swamps 29.2 (26.4–36.3) and others (Mann–Whitney, *p* < 0.001) (Figures 1(a) and 1(b)).

To assess whether this difference was significant, we performed a linear logistic regression analysis. The regression model performed well with an adjusted *R* square of 0.74. The variable “Food Swamps and Deserts” was significantly and independently associated with increased obesity prevalence (*p*=0.008). Due to a significant degree of correlation between variables, we performed collinearity statistics. The tolerance was 0.952; the variance inflation factor of 1.05 indicated an absence of collinearity impact for the variable.

## 4. Discussion

The epidemic of obesity in this country and others is expected to worsen as more people globally have increased access to processed, high-calorie food. Obesity has surpassed smoking as the number one cause of preventable disease and disability with over 230 comorbidities associated with obesity [29]. The negative health outcomes from obesity contribute to increased mortality risk from all causes and cardiovascular mortality in patients with a BMI > 25 kg/m^2^ along with increased cancer risk [30–36]. Aside from the myriad physical health outcomes, obesity also affects mental health and psychosocial functioning as individuals are frequently met with disapproval not only in the healthcare system but also in their work and school. On a larger scale, the societal and financial impact of treating obesity and dealing with its consequences is enormous. In addition to direct health care expenses, lost work productivity and lower household income cost the United States $1.4 trillion in 2017 [36–38]. Thus, both on an individual and societal level, obesity exacts an enormous cost and requires multifactorial interventions.

Although the direct contributors to obesity are straightforward, namely, sedentary behaviors and increased caloric intake, the risk factors that underlie the development of obesity are complex. Psychosocial influences, cultural norms, and myriad social determinants of health all add to the epidemic of obesity with multiple large cross-sectional epidemiological studies demonstrating an association between obesity and mental health disorders [39, 40].

Our study suggests an association between several behavioral health activities such as healthy sleep patterns, avoidance of smoking, and exercise and BMI. It also shows a stronger correlation of food swamps with high BMI than food deserts. Given the 23.5 million Americans living in food deserts, addressing equitable food access could have significant implications for public health [38, 41, 42]. Specifically, appropriating resources to disadvantaged communities can foster healthier lifestyles since such dietary behaviors are influenced by the food environment [16, 43, 44]. For example, BMI has been shown to improve when families move to more affluent neighborhoods [38].

In those areas with lower food environment quality, rates of unhealthy behavior tend to be higher. The correlation across three different unhealthy behaviors (smoking, sleeping <7 hours, and no leisure-time physical activity) suggests that unhealthy behaviors tend to cluster. There are also known pathophysiologic pathways for obesity with, for example, poor sleep through decreased leptin, increased ghrelin, and other mediators (glucagon-like peptide-1 and peptide YY) [4, 45, 46].

There could be additional factors outside those studied that also impact obesity and unhealthy behaviors such as adverse childhood events, economic instability, or threats of violence. While each patient must be approached as an individual, this population-level data suggest that unhealthy behaviors are correlated and, when evaluating obese patient, clinicians should consider other risks as well as the characteristics of their community. A corollary that should be further studied is the impact on smoking, sleep, and physical activity when healthy food stores are brought to a community. In raising the quality of the food environment, overall population health could be improved beyond a decrease in the obesity rate. While interventions to increase access to healthy food are foundational, it is valuable to also consider other factors that may work synergistically to improve health outcomes. Social relationships contribute to similar weights among a population, suggesting that shared environments, social selection, and social influence may all interact to create shared experiences and weight outcomes. Thus, clustering of obesity may also be the result of sociocultural factors.

Although obesity and mRFEI are only weakly correlated in our study, efforts to improve the food environment remain a potentially impactful avenue for addressing the growing obesity epidemic. Because the food environment tends to be poorer in low-SES communities, issues of availability cannot be addressed without also addressing accessibility and food security. Providing healthy, inexpensive whole foods is only one piece of an extremely complicated puzzle in addressing health among poorer communities but locating supermarkets in low access areas has the potential for exerting an outsized effect. One way to address this issue is to increase availability of healthy food options through community gardens, farmers' markets, and other initiatives. Additionally, policies such as zoning regulations and tax incentives can encourage grocery stores and other retailers to open in lower-income areas.

One of the limitations of our research is that as a cross-sectional analysis of secondary data sources, it can provide a general correlation at the population level. However, we cannot isolate other factors that may additionally impact obesity rates. A second limitation is that different food retailers may be opening and closing as communities evolve. The mRFEI measure uses US Census Bureau data from 2010, while population health data came from the 2018 BRFSS questionnaire. The BRFSS, conducted by phone interviews, can result in biased information since the survey relies on self-assessment and individuals may report their weight as less and their height as taller than in actuality. The mRFEI score has utility but is limited in its ability to distinguish between food deserts and food swamps and to more specifically describe the details of a food environment. There is similarly a lack of evidence of an ideal mRFEI score. Additionally, as alluded to, the factors contributing to obesity likely cannot be limited to a survey, and a complex multisystem approach is needed to appreciate the landscape of influences affecting the obesity epidemic. Certainly, the factors involved in the development of obesity must be approached individually with each unique patient, but we can still advocate for health food environments that are in the best interest of our patients in general and for equitable food options for all.

## 5. Conclusions

Our research demonstrates the interplay between food availability, specific behavioral measures, and obesity. It is important to address food inequality to create healthier and more equitable communities. Improving the food environment could specifically mean restricting the number of fast-food restaurants and convenience stores permitted to open in a given area, encouraging supermarkets, health food stores, and large grocery stores to open in food deserts, as well as ensuring that healthy food options are available at existing retailers. Health-related behaviors should be addressed in tandem with obesity counseling as these factors might cluster. Access to healthy food options regardless of income or geography is a basic human right that can have a significant impact on health and wellbeing.

## Figures and Tables

**Table 1:** Prevalence of different food environments, obesity, and behaviors in New Jersey by county.

	mRFEI	Food deserts^*∗*^ (%)	Food swamps^*∗*^ (%)	Food deserts and swamps^*∗*^ (%)	Obesity	Smoking	LPA	Sleep
Atlantic	11 [2–17]	24.5	2	26.5	30.8 [28.3–35.4]	18.7 [16.1–21.4]	32 [27.1–40.3]	39.7 [37.2–45.6]
Bergen	10 [6–14]	8.8	7.5	16.3	21.6 [20.4–23.6]	12.8 [10.9–15.3]	25.7 [22.5–29.1]	34.8 [32.9–36.9]
Burlington	13.5 [3.5–23]	23.9	1.1	25.0	30.3 [28.2–33.5]	15.7 [13.5–17.6]	25 [22.4–29.3]	36.2 [34.6–39.3]
Camden	8 [0–14]	29	5.6	34.6	28.1 [25.3–31.8]	17.4 [14.3–21.5	27.9 [24.1–32.7]	40.4 [37.5–44.8]
Cape May	11 [0–17]	41.2	0	41.2	31.7 [29.1–33.6]	16.6 [13.4–20.3]	26.5 [23.3–30.1]	36.7 [34.8–39.1]
Cumberland	11 [6–20]	11.1	0	11.1	38.5 [36.1–42.7]	21.6 [19.4–26.1]	36 [32–41.5]	40.8 [39.8–44.4]
Essex	6 [3–10]	13.9	25.7	39.6	33.85 [24.5–38.1]	18.7 [12.2–21.6]	34.1 [23.3–38.3]	43.4 [36.9–48.6]
Gloucester	11.5 [0–25.5]	27.8	0	27.8	34.9 [33.2–36.3]	16.8 [15.4–16.6]	26.6 [25.5–28.7]	38.5 [37–39.6]
Hudson	7 [5–10]	1.5	21.4	22.9	25.3 [22.6–28.6]	16.7 [14.4–18.1]	30.4 [26.8–35.5]	38.6 [37.4–39.8]
Hunterton	21 [7.8–34.8]	15.4	0	15.4	29.2 [28.6–32.2]	13.2 [12.3–14.1]	20.6 [19.6–21.8]	34 [33.6–35.3]
Mercer	6 [2.5–12.5]	20.3	20.3	40.6	31.4 [27.6–40.2]	16.7 [12.3–22.4]	28.6 [23–31.2]	38.4 [35.2–44.5]
Middlesex	9 [5–14]	17.1	7	24.1	26 [23.4–28]	13.4 [10.9–15.4]	24.6 [22.2–27]	36.4 [34.9–37.8]
Monmouth	8 [3–17]	23.5	7.6	31.1	29.7 [28.4–32.2]	14.7 [12.7–17.1]	22.9 [20.6–26.7]	36.9 [35.4–39.2]
Morris	14 [8.5–22]	14	1.1	15.1	26.1 [24.9–27.4]	12 [10.8–14.1]	21.3 [19.3–23.3]	34.3 [33.2–36]
Ocean	8.5 [0–14]	32.1	2.8	34.9	26.7 [25.8–27.9]	16.3 [13.6–18.1]	26.6 [24–29.6]	37 [34.6–38.1]
Passaic	7 [5–13]	4.5	16.4	20.9	30.8 [25.5–39.6]	15.8 [12.5–20.9]	32.9 [24.6–43.2]	37.8 [34.1–42.8]
Salem	17 [0–20]	34.8	0	34.8	30.1 [28.9–32.6]	19.3 [18–21.5]	31.5 [28.8–36.4]	37.2 [36.2–39.9]
Somerset	10.5 [5–19]	22.9	0	22.9	27 [26.1–30.4]	12.6 [11–16]	21.7 [19.3–26.5]	36.2 [33.6–38.8]
Sussex	10.5 [5–19]	37.1	0	37.1	26.8 [25.9–27.8]	15.5 [14.2–16.6]	25.4 [23.3–27]	36.1 [35.5–37.2]
Union	8.5 [6–13.3]	8.2	10.2	18.4	26.4 [23.2–32.7]	15.3 [12.7–18.5]	29.4 [24.6–36.4]	39.2 [34.9–43.4]
Warren	16 [0–33]	30.4	0	30.4	32.2 [31.4–32.9]	16.8 [15.4–19]	25.3 [23.2–26.8]	37.7 [37.1–38.7]
All New Jersey	9 [4–15]	17.9	9.3	27.2	27.9 [24.9–32.7]	15.4 [12.5–18.6]	26.5 [22.5–32.3]	37.3 [34.9–40.4]

(^*∗*^) Percent of census tracts.

## Data Availability

Data were extracted from publicly available data: US Census Bureau https://www.census.gov/data/tables/time-series/demo/popest/2010s-national-total.html and the Behavioral Risk Factor Surveillance System https://www.cdc.gov/obesity/downloads/2_16_mrfei_data_table.xls.

## References

[1] Taber D. R., Chriqui J. F., Quinn C. M., Rimkus L. M., Chaloupka F. J. (2016). Cross-sector analysis of socioeconomic, racial/ethnic, and urban/rural disparities in food policy enactment in the United States. *Health and Place*.

[2] Zenk S. N., Mentz G., Schulz A. J., Johnson-Lawrence V., Gaines C. R. (2017). Longitudinal associations between observed and perceived neighborhood food availability and body mass index in a multiethnic urban sample. *Health Education and Behavior*.

[3] Center for Health Statistics and Informatics (2019). Behavioral Risk Factor Survey (NJBRFS). https://www.state.nj.us/health/chs/njbrfs/.

[4] St-Onge M. P. (2017). Sleep-obesity relation: underlying mechanisms and consequences for treatment. *Obesity Reviews*.

[5] Carlson A., Frazao E. (2014). Food costs, diet quality and energy balance in the United States. *Physiology and Behavior*.

[6] Ghosh-Dastidar B., Cohen D., Hunter G. (2014). Distance to store, food prices, and obesity in urban food deserts. *American Journal of Preventive Medicine*.

[7] Gordon-Larsen P. (2014). Food availability/convenience and obesity. *Advances in Nutrition*.

[8] Cooksey-Stowers K., Schwartz M. B., Brownell K. D. (2017). Food swamps predict obesity rates better than food deserts in the United States. *International Journal of Environmental Research and Public Health*.

[9] Bridle-Fitzpatrick S. (2015). Food deserts or food swamps?: a mixed-methods study of local food environments in a Mexican city. *Social Science and Medicine*.

[10] Rummo P. E., Guilkey D. K., Ng S. W. (2017). Does unmeasured confounding influence associations between the retail food environment and body mass index over time? The Coronary Artery Risk Development in Young Adults (CARDIA) study. *International Journal of Epidemiology*.

[11] Morland K., Diez Roux A. V., Wing S. (2006). Supermarkets, other food stores, and obesity: the atherosclerosis risk in communities study. *American Journal of Preventive Medicine*.

[12] Schwendler T., Shipley C., Budd N. (2017). Development and implementation: B’More healthy communities for kid’s store and wholesaler intervention. *Health Promotion Practice*.

[13] Needham C., Strugnell C., Allender S., Orellana L. (2022). Beyond food swamps and food deserts: exploring urban Australian food retail environment typologies. *Public Health Nutrition*.

[14] Gartin M. (2012). Food deserts and nutritional risk in Paraguay. *American Journal of Human Biology*.

[15] Gbenro M., Student M., Brace A. M. (2017). The relationship between food deserts, farmers’ markets, Nutrition Benefits, and health in Delaware census tracts. *Delaware Journal of Public Health*.

[16] Hager E. R., Cockerham A., O’Reilly N. (2017). Food swamps and food deserts in Baltimore City, MD, USA: associations with dietary behaviours among urban adolescent girls. *Public Health Nutrition*.

[17] Byker Shanks C., Ahmed S., Dupuis V. (2020). Perceptions of food environments and nutrition among residents of the Flathead Indian Reservation. *BMC Public Health*.

[18] Budzynska K., West P., Savoy-Moore R. T., Lindsey D., Winter M., Newby P. K. (2013). A food desert in Detroit: associations with food shopping and eating behaviours, dietary intakes and obesity. *Public Health Nutrition*.

[19] Jiao J. (2016). Measuring vulnerable population’s healthy and unhealthy food access in austin, Texas. *AIMS Public Health*.

[20] Lee I. M., Shiroma E. J., Lobelo F., Puska P., Blair S. N., Katzmarzyk P. T. (2012). Lancet Physical Activity Series Working G: Effect of physical inactivity on major non-communicable diseases worldwide: an analysis of burden of disease and life expectancy. *The Lancet*.

[21] Pinto A. J., Roschel H., de Sá Pinto A. L. (2017). Physical inactivity and sedentary behavior: overlooked risk factors in autoimmune rheumatic diseases?. *Autoimmunity Reviews*.

[22] Wilmot E. G., Edwardson C. L., Achana F. A. (2012). Sedentary time in adults and the association with diabetes, cardiovascular disease and death: systematic review and meta-analysis. *Diabetologia*.

[23] United States Census Bureau (2020). 2020 Census- census tract reference map. https://www.census.gov/geographies/reference-maps/2020/geo/2020pl-maps/2020-census-tract.html.

[24] CDC (2011). Census tract level state maps of the modified food environment index (mrfei). https://stacks.cdc.gov/view/cdc/61367.

[25] Control CfD (2013). *Prevention: Census Tract Level State Maps of the Modified Retail Food Environment index (mRFEI)*.

[26] CDC (2021). Behavioral risk factor surveillance system. https://www.cdc.gov/brfss/index.html.

[27] USDA (2011). Mapping food deserts in the united states. https://www.ers.usda.gov/amber-waves/2011/december/data-feature-mapping-food-deserts-in-the-us/.

[28] WHO (2017). Obseity. https://www.who.int/health-topics/obesity/#tab=tab_1.

[29] Rueda-Clausen C. F., Ogunleye A. A., Sharma A. M. (2015). Health benefits of long-term weight-loss maintenance. *Annual Review of Nutrition*.

[30] McTigue K., Larson J. C., Valoski A. (2006). Mortality and cardiac and vascular outcomes in extremely obese women. *JAMA*.

[31] Aune D., Sen A., Prasad M. (2016). BMI and all cause mortality: systematic review and non-linear dose-response meta-analysis of 230 cohort studies with 3.74 million deaths among 30.3 million participants. *BMJ*.

[32] Falagas M. E., Kompoti M. (2006). Obesity and infection. *The Lancet Infectious Diseases*.

[33] Tsai A. G., Wadden T. A. (2013). Obesity and serious infections. *Annals of Internal Medicine*.

[34] Prospective Studies C., Whitlock G., Lewington S. (2009). Body-mass index and cause-specific mortality in 900 000 adults: collaborative analyses of 57 prospective studies. *The Lancet*.

[35] Steele C. B., Thomas C. C., Henley S. J. (2017). Vital signs: trends in incidence of cancers associated with overweight and obesity - United States, 2005-2014. *MMWR Morb Mortal Wkly Rep*.

[36] Weighing down America (2020). The health and economic impact of obesity. https://milkeninstitute.org/report/weighing-down-america-health-and-economic-impact-obesity.

[37] Narbro K., Agren G., Jonsson E., Naslund I., Sjostrom L., Peltonen M. (2002). Swedish Obese Subjects Intervention S: pharmaceutical costs in obese individuals: comparison with a randomly selected population sample and long-term changes after conventional and surgical treatment: the SOS intervention study. *Archives of Internal Medicine*.

[38] Tulane University School of Social Work (2018). Food deserts in america. https://socialwork.tulane.edu/blog/food-deserts-in-america.

[39] Chu J. M. R. (2020). *Psychosocial Factors and Outcomes of Obesity*.

[40] Nieman P., Leblanc C. M. (2012). Canadian paediatric society HAL, sports medicine C: psychosocial aspects of child and adolescent obesity. *Paediatrics and Child Health*.

[41] Kraft A. N., Thatcher E. J., Zenk S. N. (2020). Neighborhood food environment and health outcomes in US low-socioeconomic status, racial/ethnic minority, and rural populations: a systematic review. *Journal of Health Care for the Poor and Underserved*.

[42] Fitzpatrick K. G.-S. N., Greenhalgh-Stanley N., Ver Ploeg M. (2016). The impact of food deserts on food insufficiency and SNAP participation among the elderly. *American Journal of Agricultural Economics*.

[43] Miles R., Wang Y., Johnson S. B. (2018). Neighborhood built and social environments and change in weight status over the summer in low-income elementary school children. *International Journal of Environmental Research and Public Health*.

[44] Trude A. C. B., Kharmats A. Y., Hurley K. M., Anderson Steeves E., Talegawkar S. A., Gittelsohn J. (2016). Household, psychosocial, and individual-level factors associated with fruit, vegetable, and fiber intake among low-income urban African American youth. *BMC Public Health*.

[45] Spiegel K., Tasali E., Penev P., Cauter E. V. (2004). Brief communication: sleep curtailment in healthy young men is associated with decreased leptin levels, elevated ghrelin levels, and increased hunger and appetite. *Annals of Internal Medicine*.

[46] St-Onge M. P., O’Keeffe M., Roberts A. L., RoyChoudhury A., Laferrere B. (2012). Short sleep duration, glucose dysregulation and hormonal regulation of appetite in men and women. *Sleep*.

